# [Retracted] Ursolic acid suppresses the biological function of osteosarcoma cells

**DOI:** 10.3892/ol.2023.13901

**Published:** 2023-06-06

**Authors:** Yi Pei, Yueyan Zhang, Ke Zheng, Guanning Shang, Yuming Wang, Wei Wang, Enduo Qiu, Xiaojing Zhang

Oncol Lett 18: 2628–2638, 2019; DOI: 10.3892/ol.2019.10561

Following the publication of this paper, it was drawn to the Editor's attention by a concerned reader that the β-actin western blotting data featured in Fig. 4F on p. 2635 had already been published in another article written by different authors at different research institutes.

Owing to the fact that the contentious data in the above article had already been published prior to its submission to *Oncology Letters*, the Editor has decided that this paper should be retracted from the Journal. The authors were asked for an explanation to account for these concerns, but the Editorial Office did not receive a satisfactory reply. The Editor apologizes to the readership for any inconvenience caused.

